# The complete chloroplast genome sequence of *Amentotaxus yunnanensis* (Taxaceae)

**DOI:** 10.1080/23802359.2019.1637293

**Published:** 2019-07-12

**Authors:** Jun Zhang, Ze-Peng Cao, Shuo Li, Meng-Meng Liu, Xiao-Wei Huo

**Affiliations:** a College of Pharmaceutical Science, Key Laboratory of Pharmaceutical Quality Control of Hebei Province, Hebei University, Baoding, China;; b College of Traditional Chinese Medicine, Hebei University, Baoding, China;; c Institute of Bioinformatics and Medical Engineering, School of Electrical and Information Engineering, Jiangsu University of Technology, Changzhou, China

**Keywords:** *Amentotaxus yunnanensis*, chloroplast genome, mutation, phylogenetic analysis

## Abstract

The complete chloroplast genome sequence of *Amentotaxus yunnanensis* has been defined in this study. The genome is 138,604 bp in length and one of the large inverted repeats found till date. The overall GC content of the genome is 35.1%. The *A. yunnanensis* chloroplast genome contains 118 unique genes, including 81 protein-coding genes, 31 tRNA genes, and 4 rRNA genes. Nine protein-coding genes and 6 tRNA contain a single intron, while another species (*ycf3*) has a couple of introns. A neighbor-joining phylogenetic analysis suggested that *A. yunnanensis* is closely related to *A. argotaenia* and *A. formosana* within the Taxaceae family.

*Amentotaxus yunnanensis* Li belongs to Taxaceae family of conifers that is confined to southwest China, growing in limestone habitats. Demographically, the population of *A. yunnanensis* remains in small, isolated fragments, primarily with fewer than 100 individuals. Primarily, as a result of deforestation and habitat conversions, it has been listed as vulnerable (VU) by the IUCN and as a second-class national-protected plant in China (Li SH et al. 2003). In order to improve *A. yunnanensis* species management and conservation, the complete chloroplast genome of *A. yunnanensis* has been defined in this project.

Fresh leaves of *A. yunnanensis* were collected from Xiajinchang Village, Malipo County, Yunnan Province. The plant sample was taxonomically identified by Professor Yao-Hai Yang, Forestry Bureau of Xichou County, Yunnan Province. A voucher specimen (No. SHS-03-0418) was also deposited at the herbarium of Hebei University, Baoding, China. High-quality genomic DNA was extracted using DN38 DNA plant mini kit (Aidlab, Beijing, China) following the manufacturer’s instructions.

The Illumina MiSeq sequencing platform (Majorbio Biotech, Shanghai, China) was used to sequence the complete chloroplast genome of *A. yunnanensis*. We assembled the chloroplast genomes of *A. yunnanensis* with *A. argotaenia* (GenBank: KR780582) (Li J et al. 2016) as the reference using MITObim v1.8 (Hahn et al. 2013). The online tool (DOGMA) was used to annotate the cp genome of *A. yunnanensis* (Wyman et al. 2004). The annotation results were checked and corrected manually, and codon positions were determined by comparing with homologous genes from various chloroplast genomes present in the database.

The complete cp genome of *A. yunnanensis* is a circular molecule of 138,604 bp in length (GenBank accession NO. MH822838). The cp genome of *A. yunnanensis* is consistent with other sequenced cp genomes of conifers, except the absence of one of the large inverted repeats (IRs). The overall GC content f *A. yunnanensis* cp genome is 35.1%. The *A. yunnanensis* cp genome encodes a total of 118 genes, including 81 protein-coding gene, 4 rRNA genes, and 31 individual tRNA genes. Nine protein-coding genes and six tRNAs contain a single intron, while another one species (*ycf3*) has a couple of introns.

In this project, the phylogenetic analysis was performed using 77 protein-coding genes of 20 angiosperms (Figure 1). MAFFT v7.017 was used to align the 77 protein-coding gene sequences(Katoh et al. 2017). Phylogenetic analysis was conducted based on neighbor-joining method with the default parameters in MEGA 7.0 (Kumar et al. 2016). The bootstrap values next to the branches are based on 1000 resamplings. All 20 species were clustered into two monophyletic groups, corresponding to the three subfamilies Taxaceae, Cupressaceae, and Sciadopityaceae. The phylogenetic tree strongly supports a sister relationship between *Amentotaxus* and *Taxus.*

**Figure 1.**
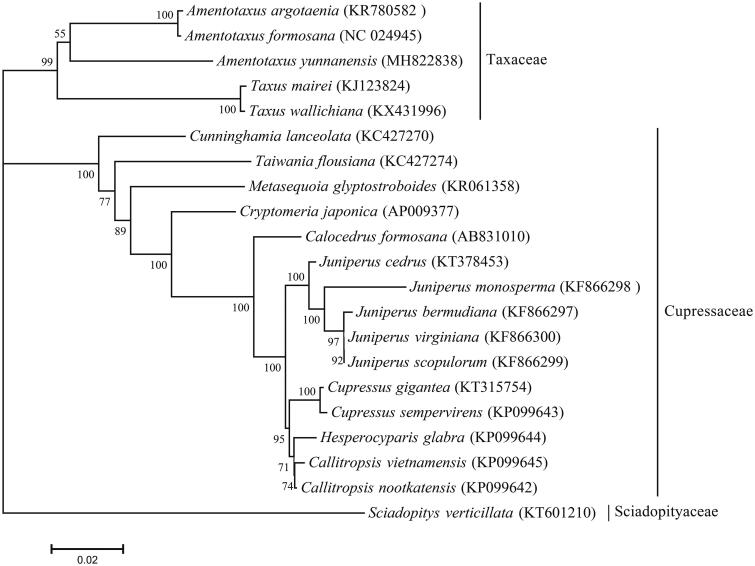
Phylogenetic relationships of 20 Pinidae species based on the neighbor-joining (NJ) analysis. The bootstrap values were based on 1000 replicates, and are shown next to the branches.
